# Influence of intraoperative vasopressor use on indocyanine green fluorescence angiography: first evaluation in an experimental model

**DOI:** 10.1038/s41598-021-89223-2

**Published:** 2021-05-06

**Authors:** Mahdi Al-Taher, Tim Pruimboom, Rutger M. Schols, Nariaki Okamoto, Nicole D. Bouvy, Laurents P. S. Stassen, René R. W. J. van der Hulst, Michael Kugler, Alexandre Hostettler, Eric Noll, Jacques Marescaux, Sophie Diemunsch, Michele Diana

**Affiliations:** 1 IRCAD, Research Institute Against Digestive Cancer, 1, Place de l’Hôpital, 67000 Strasbourg, France; 2 Department of Surgery, Maastricht University Medical Center, Maastricht, The Netherlands; 3 NUTRIM School of Nutrition and Translational Research in Metabolism, Maastricht University Medical Center, Maastricht University, P. Debyelaan 25, 6229 HX Maastricht, The Netherlands; 4 Department of Anesthesiology, Critical Care and Prehospital Emergency Medicine, University Hospital of Strasbourg, Strasbourg, France; 5 Department of General, Digestive, and Endocrine Surgery, University Hospital of Strasbourg, Strasbourg, France; 6 ICube Laboratory, Photonics Instrumentation for Health, Strasbourg, France

**Keywords:** Gastrointestinal models, Imaging and sensing

## Abstract

Intraoperative indocyanine green (ICG) fluorescence angiography has gained popularity and acceptance in many surgical fields for the real-time assessment of tissue perfusion. Although vasopressors have the potential to preclude an accurate assessment of tissue perfusion, there is a lack of literature with regards to its effect on ICG fluorescence angiography. An experimental porcine model was used to expose the small bowel for quantitative tissue perfusion assessment. Three increasing doses of norepinephrine infusion (0.1, 0.5, and 1.0 µg/kg/min) were administered intravenously over a 25-min interval. Time-to-peak fluorescence intensity (TTP) was the primary outcome. Secondary outcomes included absolute fluorescence intensity and local capillary lactate (LCL) levels. Five large pigs (mean weight: 40.3 ± 4.24 kg) were included. There was no significant difference in mean TTP (in seconds) at baseline (4.23) as compared to the second (3.90), third (4.41), fourth (4.60), and fifth ICG assessment (5.99). As a result of ICG accumulation, the mean and the maximum absolute fluorescence intensity were significantly different as compared to the baseline assessment. There was no significant difference in LCL levels (in mmol/L) at baseline (0.74) as compared to the second (0.82), third (0.64), fourth (0.60), and fifth assessment (0.62). Increasing doses of norepinephrine infusion have no significant influence on bowel perfusion using ICG fluorescence angiography.

## Introduction

Fluorescence angiography, using indocyanine green (ICG) as a contrast agent, is increasingly applied by surgeons during surgical procedures, facilitating intraoperative decision-making. This imaging technique is fast (i.e., a matter of seconds to minutes), safe, and easy to perform, and multiple assessments can be performed during a single procedure^1^. Consequently, ICG fluorescence angiography is gaining popularity and is meeting acceptance in many surgical fields^2–5^, including colorectal surgery^6,7^.

Anastomotic leakage (AL) is one of the dreaded complications in colorectal surgery, with an incidence of up to 20% of cases^6,8^. It has been associated with an increased postoperative morbidity and mortality, and even when managed, it leads to a prolonged hospital stay and to increased healthcare costs^7,8^. As adequate perfusion is essential for optimal healing and AL prevention, an insufficient blood supply at the proximal or distal end of the anastomosis is one of several factors which have been associated with a greater risk of AL in case of an intraoperatively “water-tight” anastomosis. Accordingly, the assessment of bowel perfusion and intraoperative modification of the level of resection or anastomosis in case of insufficient perfusion may contribute to a reduced risk of AL^7^.

Traditionally, bowel perfusion is evaluated by the surgeon through a direct visualization of the anastomosis, including the evaluation of the serosal and mucosal color, bowel peristalsis, and pulsation of mesenteric arteries or bleeding at the cut edge of the bowel^7,8^. However, these subjective signs did not allow to evaluate microperfusion and were found to be unreliable since the accuracy of AL prediction by surgeons was low^7–9^. For this reason, ICG fluorescence angiography was proposed as an objective imaging technique which allows for the real-time assessment of bowel perfusion. After intravenous administration, ICG is bound to plasma protein. When exposed to near-infrared excitation, it re-emits a fluorescent light. Bowel perfusion may be quantified by using fluorescence intensity, which is proportional to bowel vascularization^10^. Recent systematic reviews demonstrated that ICG fluorescence angiography seems to reduce AL rates as compared to conventional techniques in colorectal surgery^7,11^. Liu et al. reported an AL rate of 3.8% in the ICG group as compared to 7.8% in the non-ICG group in a meta-analysis including a total of 4,037 patients^12^.

Nevertheless, the evaluation of fluorescence intensity remains a static measure with no consideration of ICG diffusion over time. Consequently, a dynamic fluorescence videography technique, which integrates near-infrared endoscopy and specific software called fluorescence-based enhanced reality (FLER), has been developed^13^. Dynamic fluorescence angiography allows for time-to-peak fluorescence intensity (TTP). It is found to be a promising tool for the real-time imaging of bowel perfusion in an easy and accurate way^10,14^. Additionally, FLER analyses were found to be correlated with local capillary lactate (LCL) levels, in the experimental^10^ and clinical setting^15^.

While vasopressors (e.g., norepinephrine) are often used during surgery to restore and maintain blood pressure levels in case of hypotension^16^, these substances have the potential to drastically reduce blood flow via vasoconstriction. Consequently, they may potentially preclude an accurate assessment of tissue perfusion when using ICG fluorescence angiography^17,18^.

To date, there is a limited number of studies which evaluated the effect of vasopressors on tissue perfusion assessment using ICG fluorescence angiography. The aim of this study was to investigate the effect of increasing doses of norepinephrine on bowel perfusion assessment using ICG fluorescence angiography in a porcine intestinal model.

## Methods

This study, which is part of the Endoscopic Luminescent Imaging for Oncology Surgery (ELIOS) project, was performed according to the National Institutes of Health guidelines for the use of experimental animals, respecting the ARRIVE guidelines^19^. 

### Norepinephrine infusion

Before finalizing the study protocol, consideration was given to use either increasing boluses of norepinephrine or an increasing continuous norepinephrine infusion. Norepinephrine is a sympathomimetic amine with a primarily agonistic effect at alpha-1 and beta-1 receptors, which increases systemic vascular resistance and potentially increases cardiac output respectively^20^. Due to a short half-life of 2.5 min, an intravenous bolus injection of norepinephrine would only induce short-term hypertension and tachycardia. In this period of time, ICG fluorescence angiography could be performed. For this evaluation, and for ethical purposes, respecting the ‘3R’ (replace, refine, reduce) principles of animal research^21^, two pilot pigs used for educational purposes without causing any intestinal damage, were utilized to assess the effect of increasing doses of bolus injections versus increasing doses of continuous infusion of norepinephrine. In the ‘bolus injection pig’, a very short time period of vasopressor effect was observed as blood pressure and heart rate dropped within approximately two minutes during ICG fluorescence angiography assessment. In the ‘continuous infusion pig’, no decrease was observed as blood pressure and heart rate were maintained during infusion. For this logistical reason, it was decided to use increasing doses of continuous norepinephrine infusion in this study. Additionally, it was decided to use the following doses of norepinephrine of 0.1, 0.5, and 1.0 µg/kg/min, since these doses resulted in a relevant increase in blood pressure and are within the range of doses frequently used in the clinical setting.

### Animal preparation

A total of five adult female large white pigs (*Sus scrofa domesticus)* were included. According to the design of the study, every animal served as its own internal control. All animals were fasted for 24 h with free access to water. A qualified anesthesiologist (EN and SD) performed every anesthesia-related steps of the procedure.

The animals were sedated with an intramuscular injection of zolazepam and tiletamine 10 mg/kg (Zoletil ND, Virbac, France). After general anesthesia induction using an intravenous administration of propofol 3 mg/kg (Propofol Lipuro ND, B Braun, France) and rocuronium 0.8 mg/kg (Esmeron ND, MSD, France), the animals were intubated and mechanically ventilated in a supine position. Anesthesia was maintained by means of a continuous inhalation of isoflurane 2% (Isoflurin ND, Axience, France) and a mixture of 50% nitrous oxide in oxygen. The dose of anesthetics was increased when necessary according to the reflex status of the animals (i.e., palpebral reflexes and jaw tone). Intravascular injections of buprenorphine (Buprecare ND, Axience, France) 0.01 mg/kg were used for analgesia. All animals were infused with Ringer’s lactate intravenously at a rate of 4 mL/kg/h.

**Figure 1:**
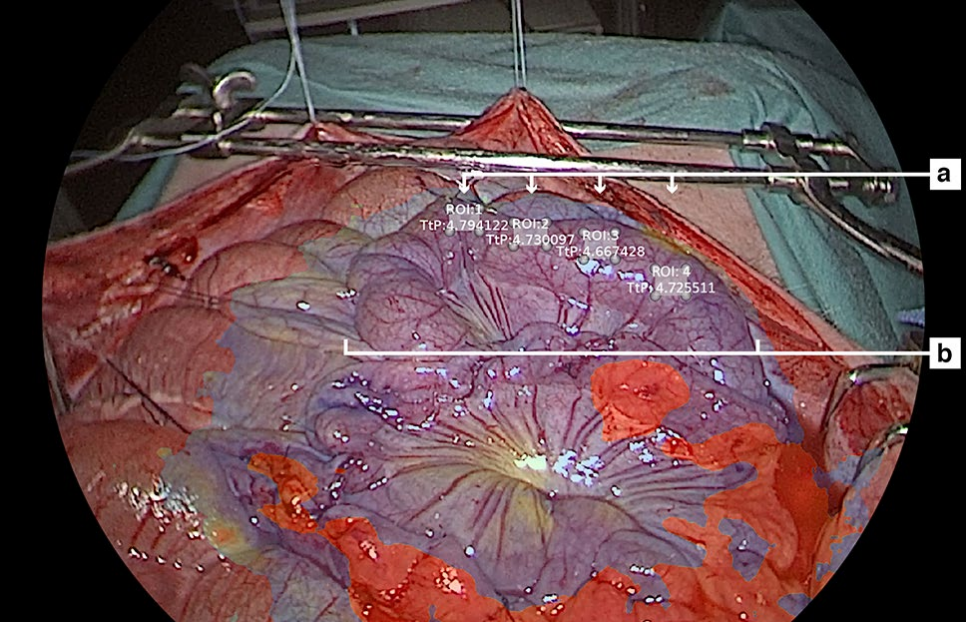
Study set-up with fluorescence-based enhanced reality (FLER) overlay. (**a)** Randomly chosen regions of interest (ROIs) and (**b)** small intestine parts under investigation.

**Figure 2:**
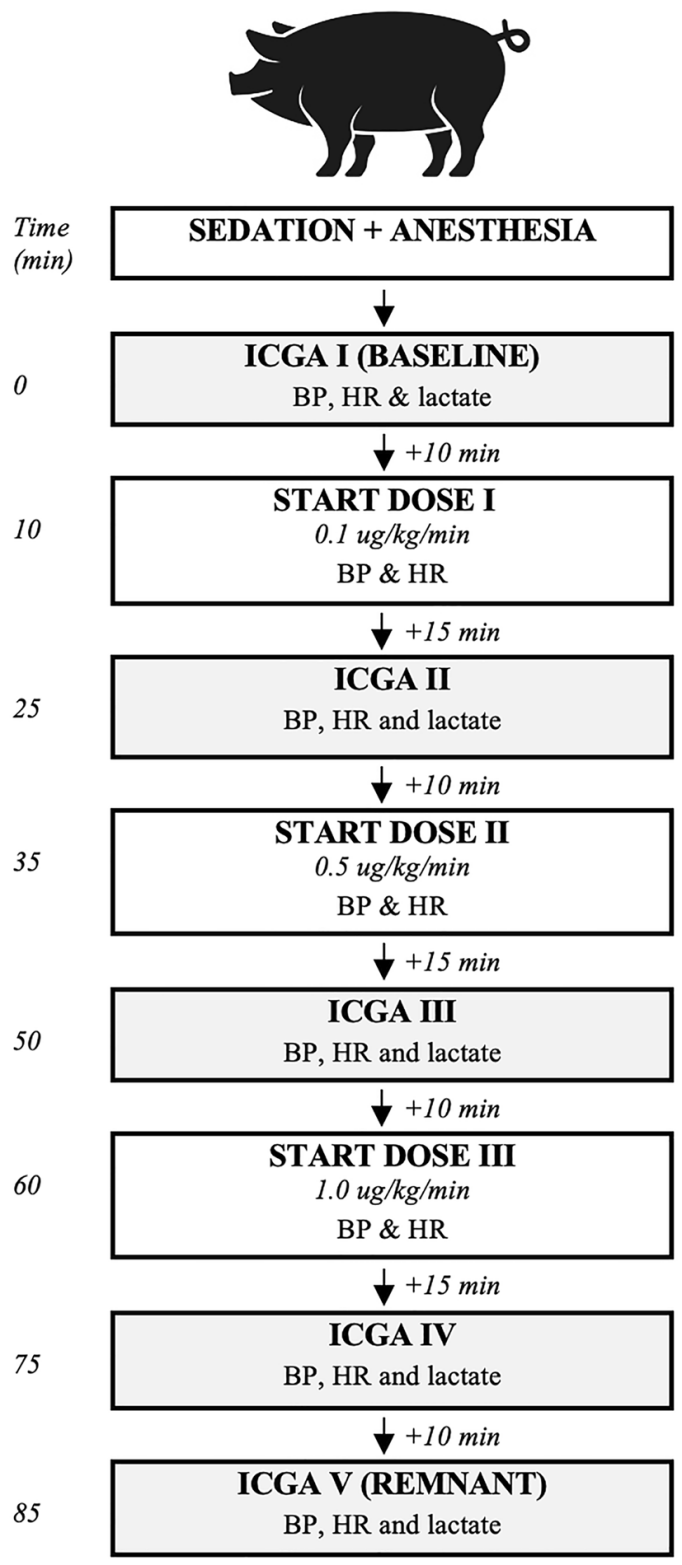
Study flowchart. *ICGA* indocyanine green angiography, *BP* blood pressure,* HR* heart rate.

A midline laparotomy was performed to expose the small bowel (Fig. 1 for the study set-up). A 30 cm segment of the small intestine (250 cm from the pylorus) was loosely fixed to both sides of the abdominal wall with Vicryl sutures to guarantee bowel exposure during this study. No further bowel manipulation was performed. During the protocol, three increasing doses of continuous norepinephrine infusion (0.1, 0.5, and 1.0 µg/kg/min) were administered intravenously over a 25-min interval (Fig. 2 for the study flowchart). Heart rate and blood pressure were continuously monitored through an arterial line inserted into the femoral artery. At the end of the protocol, pigs were sacrificed under deep anesthesia (isoflurane 5%) with intravenous administrations of pentobarbital 40 mg/kg (Exagon ND, Axience, France).

### ICG fluorescence angiography

In this study, an ICG fluorescence videography system integrated within a near-infrared endoscope (KARL STORZ GmbH & CO. KG, Germany) was used. It could detect the ICG-emitted fluorescence signal. After exposing the small bowel, the camera was fixed to an articulated arm in order to stabilize the image and allow for repetitive assessments (e.g., ensuring a stable distance and angle during ICG fluorescence angiography assessments). In a recent analysis of the European registry on fluorescence image-guided surgery (EURO-FIGS), an ICG dose of 0.1 to 0.2 mg/kg was identified as the most frequently used clinical dose for near-infrared fluorescence angiography^22^. To standardize the method of ICG administration, a bolus of ICG (Infracyanine, Laboratoires Serb, Paris, France) at a dose of 0.1 mg/kg was administered intravenously through a central venous catheter in the right internal jugular vein (Vygon, arterial LeaderCath, 6 French, 15 cm).

The ICG-emitted fluorescence signal was analysed using the ER-PERFUSION software (IRCAD, Strasbourg, France), which allowed for a virtual perfusion cartography based on time-to-peak fluorescence intensity (TTP; in seconds). TTP results from the velocity of the fluorescence signal until it reaches its maximum intensity peak in a specific region of interest (ROI) within the first 40 s following ICG injection^23^. This is a relatively short period following ICG injection, as a result of ICG administration via the central venous catheter. The virtual perfusion cartography is subsequently overlapped onto the images, providing fluorescence-based enhanced reality (FLER). In this study, TTP was measured from the time when there was 25% of fluorescence intensity to 75% of fluorescence intensity using the ER-PERFUSION software. In addition, the minimum and maximum absolute fluorescence intensities were measured in four randomly chosen ROIs on the bowel surface using the FLER software. TTP and absolute fluorescence intensity were analysed as mean of the four ROIs, together with the mean difference in maximum and minimum absolute fluorescence intensities.

### Capillary lactate levels

Local bowel capillary lactate (LCL) levels were measured in the blood using an EDGE lactate analyser (ApexBio, Taipei, Taiwan, People’s Republic of China) by puncturing the bowel serosa in the chosen ROI with a needle. LCL levels were obtained during all five fluorescence videography assessments.

### Statistical analysis

Continuous variables are presented as means with standard deviation (SD). Categorical data were reported as frequency and proportion. Fisher’s exact test was used to calculate *P* values for categorical variables. The paired sample T-test was used to analyse the differences in TTP fluorescence intensity (in seconds) and mean absolute fluorescence intensity in ROIs between the different vasopressor doses. All results were analysed using IBM SPSS Statistics for Windows, version 24 (IBM Corporation, Armonk, NY, USA). A *p* value < 0.05 was considered statistically significant.

### Ethics approval

The protocol was approved by the local Ethical Committee on Animal Experimentation (ICOMETH No. 38.2020.02.003) and by the French Ministry of Superior Education and Research (MESR) under the following reference: APAFIS#8721-2017013010316298-v2. All animals used in the experimental laboratory were managed according to French laws for animal use and care and according to the directives of the European Community Council (ECC).

## Results

The included animals had a mean weight of 40.3 ± 4.24 kg (range: 33.0 to 44.0 kg). During the protocol, all pigs underwent five ICG fluorescence angiographies, namely once at baseline, three times following increasing doses (0.1, 0.5, and 1.0 µg/kg/min) of norepinephrine infusion, and once following discontinuation of norepinephrine infusion. In general, blood pressure and heart rate increased gradually as the infusion dose increased. After discontinuation of norepinephrine infusion, a substantial decrease in blood pressure and heart rate was observed (Tables 1 and 2).

**Table 1:** Measurements (BP, HR, and lactate levels) per pig over time.

	Pig 1	Pig 2	Pig 3	Pig 4	Pig 5
Weight (kg)	33.0	42.1	41.4	41.0	44.0
ICG per dose (mg)	3.3	4.2	4.1	4.1	4.4
**0 min: ICGA 1 (baseline)**					
Systolic BP (mmHg)	85	53	58	60	52
Diastolic BP (mmHg)	48	38	30	35	29
HR (bpm)	97	90	84	63	95
Lactate (mmol/L)	0.8	Low^a^	0.6	1.1	Low^a^
**10 min: start first NE dose**					
Systolic BP (mmHg)	66	57	61	55	35
Diastolic BP (mmHg)	38	41	32	33	27
HR (bpm)	92	89	88	60	83
**25 min: ICGA II (first dose)**					
Systolic BP (mmHg)	89	76	91	93	70
Diastolic BP (mmHg)	60	58	48	57	38
HR (bpm)	95	76	88	82	98
Lactate (mmol/L)	Low^a^	Low^a^	Low^a^	1.3	1.0
**35 min: start second NE dose**					
Systolic BP (mmHg)	94	65	78	88	64
Diastolic BP (mmHg)	51	50	41	55	33
HR (bpm)	98	79	90	82	98
**50 min: ICGA III (second dose)**					
Systolic BP (mmHg)	155	77	130	134	89
Diastolic BP (mmHg)	97	52	60	102	46
HR (bpm)	101	95	136	105	107
Lactate (mmol/L)	0.6	Low^a^	Low^a^	Low^a^	0.8
**60 min: start third NE dose**					
Systolic BP (mmHg)	128	49	101	124	80
Diastolic BP (mmHg)	68	38	47	73	42
Heart rate (bpm)	92	103	125	103	125
**75 min: ICGA IV (third dose)**					
Systolic BP (mmHg)	127	125	128	132	81
Diastolic BP (mmHg)	65	74	62	83	45
HR (bpm)	92	146	143	120	142
Lactate (mmol/L)	Low^a^	Low^a^	Low^a^	0.6	0.6
**85 min: ICGA V (remnant)**					
Systolic BP (mmHg)	48	32	30	45	41
Diastolic BP (mmHg)	32	23	20	25	24
HR (bpm)	125	113	116	76	110
Lactate (mmol/L)	Low^a^	Low^a^	Low^a^	Low^a^	0.7

*ICGA* indocyanine green angiography, *BP* blood pressure, *HR* heart rate, *bpm* beats per minute, *NE* norepinephrine.

^a^Lactate levels between 0.1 and 0.6 mmol/L.

**Table 2:** Mean measurements over time.

	0 min ICGA I	10 min	25 min ICGA II	35 min	50 min ICGA III	60 min	75 min ICGA IV	85 min ICGA V
NE dose (µg/kg/min)	0	0	0.1	0.1	0.5	0.5	1.0	1.0
HR (bpm), mean SD	86 ± 14	82 ± 13	88 ± 9	89 ± 9	109 ± 16	110 ± 15	129 ± 23	108 ± 19
Systolic BP (mmHg), mean SD	62 ± 14	55 ± 12	84 ± 10	78 ± 13	117 ± 33	96 ± 33	119 ± 21	39 ± 8
Diastolic BP (mmHg), mean SD	36 ± 8	34 ± 6	52 ± 9	46 ± 9	71 ± 26	54 ± 16	66 ± 14	25 ± 4
Lactate range (mmol/L)	Low—0.83	NE	Low—1.15	NE	Low—0.70	NE	Low—0.60	Low—0.70

*ICGA* indocyanine green angiography (I = baseline, II = after norepinephrine (NE) dose 1, III = after NE dose 2, IV = after NE dose 3, V = remnant measurement following discontinuation of NE), *HR* heart rate,* BP* blood pressure, *bpm* beats per minute*.*

### Time-to-peak fluorescence intensity (TTP)

**Figure 3:**
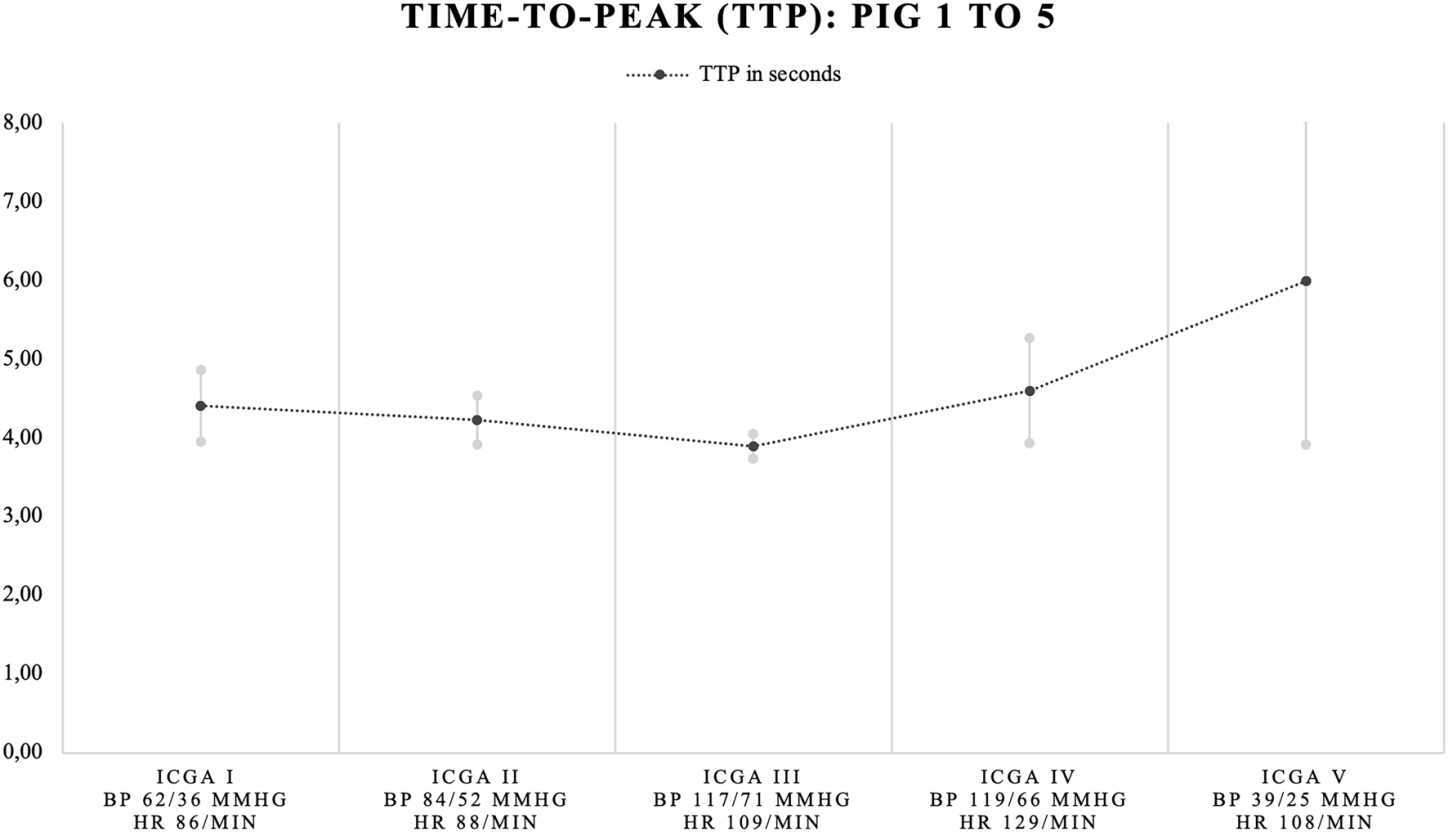
Mean time-to-peak (TTP) in seconds for all 5 pigs. Mean TTP (in black) with standard deviations (in grey). X-axis: ICGA assessment number, mean BP and HR for all pigs. Y-axis: mean TTP in seconds. *ICGA* indocyanine green angiography (I = baseline, II = after norepinephrine (NE) dose 1, III = after NE dose 2, IV = after NE dose 3, V = remnant measurement following discontinuation of NE), BP = blood pressure, HR = heart rate.

Mean TTP of the four regions of interest after the first (4.23 ± 0.30 s) and second dose of norepinephrine (3.90 ± 0.16 s) were slightly lower as compared to baseline (4.41 ± 0.46 s) assessment (− 0.18 and − 0.51 s respectively). Conversely, mean TTP after the third dose (4.60 ± 0.68 s) and after discontinuation of norepinephrine infusion (5.99 ± 2.07) were higher as compared to baseline assessment (+ 0.19 and + 1.58 respectively) (Fig. 3). However, mean TTP did not significantly differ from baseline assessment (Table 3). In addition, mean TTP per ROI in the first, second, third dose assessment, and mean TTP in the assessment following discontinuation of norepinephrine infusion, did not significantly differ from baseline.

**Table 3:** Difference in mean TTP in seconds over increasing infusion doses.

	TTP, mean ± SD	TTP, mean ± SD	Mean difference	P value
Baseline vs. NE dose 1	4.41 ± 0.46	4.23 ± 0.30	- 0.18	0.204
Baseline vs. NE dose 2	4.41 ± 0.46	3.90 ± 0.16	- 0.51	0.119
Baseline vs. NE dose 3	4.41 ± 0.46	4.60 ± 0.68	+ 0.19	0.555
Baseline vs. NE remnant	4.41 ± 0.46	5.99 ± 2.07	+ 1.58	0.110

*NE* norepinephrine, *ICGA* indocyanine green angiography, *TTP* time-to-peak, *SD* standard deviation.

### Absolute fluorescence intensity

**Figure 4:**
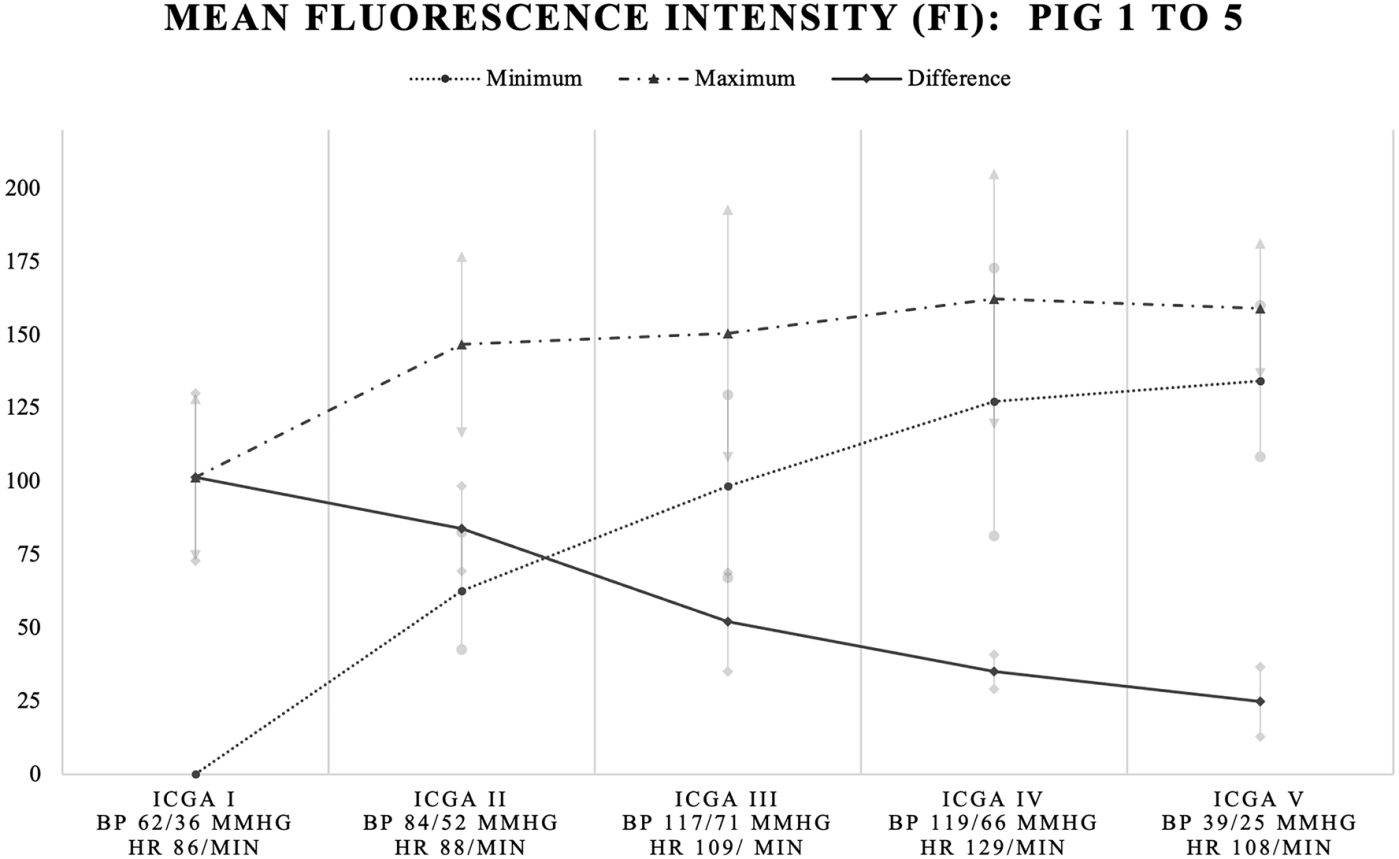
Mean minimum, maximum, and difference in absolute fluorescence intensity (FI) for all 5 pigs. Mean fluorescence intensity (in black) with standard deviations (in grey). X-axis: ICGA assessment number, mean BP and HR for all pigs. Y-axis: mean fluorescence intensity. *ICGA* indocyanine green angiography (I = baseline, II = after norepinephrine (NE) dose 1, III = after NE dose 2, IV = after NE dose 3, V = remnant measurement following discontinuation of NE), *BP* blood pressure, *HR* heart rate.

Mean minimum fluorescence intensity of the four ROIs increased during the protocol from 0 at baseline to 62.7, 98.3 (+ 57% increase as compared to previous ICG fluorescence angiography), 127.1 (+ 29%), and 134.2 (+ 6%) after the first, second, and third dose, and after discontinuation of norepinephrine infusion respectively. Mean maximum fluorescence intensity of the four ROIs also increased from 101.4 at baseline to 146.6 (+ 45%), 150.4 (+ 3%), 162.1 (+ 8%), and 159.0 (− 2%) after the first, second, and third dose, and after discontinuation of norepinephrine infusion respectively. Since the maximum intensity increased in a slower manner as compared to the minimum intensity, the mean difference decreased from 101.4 to 24.8 (Fig. 4). Mean minimum fluorescence intensity in the first (*p* = 0.002), second (*p* = 0.002), third (*p* = 0.003), and remnant assessment (*p* ≤ 0.001) were significantly different as compared to the baseline assessment, as well as the maximum fluorescence intensity (*p* = 0.011, 0.016, 0.027, and 0.002 respectively).

Regarding the mean difference in fluorescence intensity, only the mean difference between fluorescence intensity in the first dose assessment did not significantly differ from baseline (*p* = 0.208, in contrast to the second (*p* = 0.006), third (*p* = 0.011), and remnant assessment (*p* = 0.003).

### Local capillary lactate (LCL) levels

Local capillary lactate (LCL) levels at the time of the ICG fluorescence angiography assessment are presented in Table 3. LCL levels defined as ‘low’ represent a value between 0.1 and 0.59 mmol/L. In statistical analyses comparing baseline and the following ICG fluorescence angiographies, all ‘low’ LCL level results were regarded as 0.59 in order to prevent any underestimation bias. The subsequent LCL levels were 0.74, 0.82, 0.64, 0.60, and 0.62 from baseline to remnant assessment respectively. No significant difference in LCL levels was found.

## Discussion

Although there were a few previous studies which evaluated the effect of vasopressors on free flap perfusion using ICGA, this is the first study evaluating the influence of increasing doses of intraoperative vasopressor use on ICG fluorescence angiography in a standardized porcine model^24,25^. Based on this model, no difference was found in mean time-to-peak fluorescence intensity (TTP) between baseline and increasing doses of norepinephrine infusion. Additionally, a significant increase in mean minimum and maximum absolute fluorescence intensities was observed, as well as a significant decrease in mean difference between these two measurements.

In gastrointestinal surgery, an adequate intraoperative assessment of bowel perfusion is mandatory in order to prevent anastomotic leakage. Over the last decade, ICG fluorescence angiography has gained popularity for the real-time assessment of bowel perfusion^12^. Likewise, ICG fluorescence angiography is more frequently applied to plastic and reconstructive surgery to objectively evaluate flap perfusion^2^. In that surgical field, it has been suggested that pharmacologically induced vasoconstriction could preclude an accurate estimation of tissue perfusion, while an adequate regulation of systemic blood pressure is fundamental during every surgical procedure^18^. Despite a suspicion supported by anecdotal reports in the literature, no previous study reported the effect of vasopressors, such as norepinephrine, on tissue perfusion assessment using ICG fluorescence angiography.

TTP as part of fluorescence-based enhanced reality (FLER) was considered the primary outcome in the current study. The measurement corresponds to the mean time elapsed for the fluorescence signal to reach its maximum intensity in a given area^26^. TTP offers two great advantages as compared to absolute fluorescence intensity values. First, TTP is independent of the distance between the ICG camera and the ROI, whereas absolute fluorescence intensity is highly dependent on distance^1,14^. In the current study, the distance between the camera and the bowel was kept constant throughout perfusion assessments. Nonetheless, it remains relevant in clinical perfusion assessments.

Theoretically, the ICG plasma half-life of approximately 3 to 5 min allows for multiple perfusion assessments throughout a surgical procedure^1^. However, in a previous preliminary test by Diana et al. concerning a series of ICG injections (0.125 mg/kg every 15 min) while focusing on a healthy small bowel loop, ICG accumulation was observed. On the other hand, the calculated TTP remained constant in each assessment^14^. As a result, a second and major advantage of TTP is that it truly allows for multiple and repetitive assessments. Only the additional signal is interpreted and the “noise” produced by the accumulation of fluorescent dye does not affect TTP. Consequently, we derive that increasing doses of norepinephrine, as explored in this experimental porcine model, have no effect on bowel perfusion assessment using ICG fluorescence. Notably, the TTP increased from 4.60 s after dose 3 to 5.99 s after norepinephrine discontinuation (“remnant assessment”). Although this is a substantial increase, the mean difference in TTP was not significant as compared to the baseline assessment. The impaired clinical condition of the pigs, with a mean blood pressure of 39/25 mmHg at the end of the study, resulting from the termination of continuous norepinephrine infusion, contributed to this increase in TTP.

When considering absolute fluorescence intensity in the current study, an increase in mean minimum and maximum fluorescence intensity is consistent with the aforementioned study^14^. Although a 25-min interval (except between ICG fluorescence angiography assessment IV and V) was maintained to ensure ICG wash-out following the ICG injections of 0.1 mg/kg, a significant ICG accumulation was observed. Consequently, there is more reason to believe that it is better to use a dynamic fluorescence videography technique, such as FLER analysis, over absolute fluorescence intensity in case of repeat perfusion assessments throughout a surgical procedure. Notably, a decrease in the mean difference in fluorescence intensity was noted. It is likely due to approaching an absolute maximum fluorescence intensity level as a result of dye accumulation within the tissue.

Local capillary lactate (LCL) levels reflect tissue oxygenation in bowel cells, and these were previously correlated with bowel perfusion using ICG fluorescence angiography. In a previous study, it was concluded that the mean LCL level in an ischemic bowel region (5.6 ± 2.8 mmol/L) was significantly higher than LCL levels in a bowel region at 25% of perfusion on ICG fluorescence angiography (3.7 ± 1.7 mmol/L) and in a bowel region at 75% of perfusion (2.9 ± 1.3 mmol/L)^10^. In the current study, low LCL levels were observed with no significant increase. This reflects the healthy state and non-ischemic condition of the bowel under investigation.

With regards to the infusion dose of norepinephrine, the effect on beta-1 adrenergic receptors may be more distinct at low doses (less than 2 µg/min), potentially leading to an increased cardiac output. In doses higher than 3 µg/min, the alpha-1 adrenergic effect may predominate, resulting in vasocontraction^20^. Since the minimum dose was 3.3 µg/min in the current study, this should result in vasoconstriction and in a dose-dependent increase in systemic vascular resistance. Due to this particular effect of norepinephrine, plastic and reconstructive surgeons have hypothesized that vasoconstriction comprises blood flow of superficial capillaries, reducing the potential of ICG fluorescence angiography in order to accurately assess flap perfusion in reconstructive surgery^17,18^. The results of the current study suggest that there was no alteration in bowel perfusion based on ICG fluorescence angiography. This might be a result of an increase in blood pressure, which leads to a compensation for the vasoconstrictive effect of norepinephrine. This finding is supported by previous studies in which norepinephrine was found to preserve intestinal microcirculatory blood flow^27,28^ and microcirculatory flap perfusion^29,30^. Conversely, another study found that norepinephrine decreased intestinal microcirculatory blood flow, despite significantly increased arterial blood pressure^31^. However, the design of the current study is different as compared to previous studies in which laser Doppler flowmetry (LDF) was used to evaluate microcirculatory blood flow^27–31^.

Although plastic and reconstructive surgery concerns a different surgical field, the principles of perfusion assessment remain similar. Two recent clinical studies in this particular field have evaluated the effect of vasopressor use on flap perfusion using ICGA assessment^24,25^. Anker et al. concluded that norepinephrine concentrations of 0.065 ± 0.020 μg/kg/min had no clinically significant impact on microperfusion^24^. Massaro et al. concluded that changes in mean perfusion of the free flap during the intraoperative period are nominal^25^. However, in contrast to the current study, both studies were not performed in a standardized fashion with regard to the vasopressor dose. While the results of the current standardized animal study are consistent with previous findings and suggest that increasing doses of norepinephrine may also have no effect on perfusion assessment using ICG fluorescence angiography in other types of tissue (e.g., adipocutaneous or musculocutaneous flap perfusion in reconstructive procedures), this should be confirmed in future studies.

Despite the novelty of this first experimental study reporting on the effect of increasing doses of norepinephrine on tissue perfusion assessment using ICG fluorescence angiography, some limitations need to be addressed. Five pigs constitute a small study population and only female pigs were used^19^. However, multiple ICGA measurements were obtained without unreasonable outliers. Although one of the animals had a considerably lower total body weight of 33.0 kg compared with the mean weight of 40.3 kg, we believe that the difference in total body weight does not influence outcomes, as all animals act as their own control.

In addition, only healthy small intestines were investigated in this study. Although no difference was found in mean TTP between baseline and increasing doses of norepinephrine infusion in the current study, we are unsure about the results in diseased bowel areas. It is our intention to study this clinically relevant problem in a future experimental animal model with an ischemic bowel model by clamping the arterial supply of bowel segments in combination with the use of vasopressors. Furthermore, the study is limited by the absence of a control group in which ICG fluorescence angiography is performed without any administration of norepinephrine. Conversely, this study is strengthened by the use of two ‘pilot pigs’ in advance, in order to assess the effect of bolus injections versus a continuous infusion of norepinephrine. Continuous infusion was chosen over bolus injections, since an increase in blood pressure and heart rate was maintained with a continuous infusion and its administration relates more to the daily clinical practice. In addition, two previous studies concerning the effect of vasopressors on intestinal blood flow and oxygen supply also used continuous norepinephrine infusion in doses ranging from 0.01 to 2.0 µg/kg/min^28,31^.

In conclusion, increasing doses of norepinephrine (0.1, 0.2, and 0.5 µg/kg/min respectively) have no statistically significant influence on bowel perfusion assessment as time-to-peak fluorescence intensity remains constant during the FLER analysis. Secondly, ICG accumulation was observed when using absolute fluorescence intensity, which is an important finding for future studies, reflecting the need for a dynamic fluorescence videography technique over absolute fluorescence intensity in case of repeat perfusion assessments.

## Data Availability

The datasets generated during and/or analysed during the current study are available from the corresponding author upon reasonable request.
